# Decreased Expression of Innate Immunity-Related Genes in Peripheral Blood Mononuclear Cells from Patients with IgG4-Related Disease

**DOI:** 10.1371/journal.pone.0126582

**Published:** 2015-05-14

**Authors:** Akio Nakajima, Yasufumi Masaki, Takuji Nakamura, Takafumi Kawanami, Yasuhito Ishigaki, Tsutomu Takegami, Mitsuhiro Kawano, Kazunori Yamada, Norifumi Tsukamoto, Shoko Matsui, Takako Saeki, Kazuichi Okazaki, Terumi Kamisawa, Taiichiro Miyashita, Yoshihiro Yakushijin, Keita Fujikawa, Motohisa Yamamoto, Hideaki Hamano, Tomoki Origuchi, Shintaro Hirata, Hiroto Tsuboi, Takayuki Sumida, Hisanori Morimoto, Tomomi Sato, Haruka Iwao, Miyuki Miki, Tomoyuki Sakai, Yoshimasa Fujita, Masao Tanaka, Toshihiro Fukushima, Toshiro Okazaki, Hisanori Umehara

**Affiliations:** 1 Hematology and Immunology, Kanazawa Medical University, Uchinada, Ishikawa 920–0293, Japan; 2 Medical Research Institute, Kanazawa Medical University, Uchinada, Ishikawa 920–0293, Japan; 3 Division of Rheumatology, Department of Internal Medicine, Kanazawa University Hospital, Ishikawa 920–8641, Japan; 4 Department of Medicine and Clinical Science, Gunma University Graduate School of Medicine, Gunma 371-8511, Japan; 5 Health Administration Center University of Toyama, Toyama 930–0194, Japan; 6 Department of Internal Medicine, Nagaoka Red Cross Hospital, Niigata 940–2085, Japan; 7 Third Department of Internal Medicine, Division of Gastroenterology and Hepatology, Kansai Medical University, Osaka 573–1191, Japan; 8 Department of Internal Medicine, Tokyo Metropolitan Komagome Hospital, Tokyo 113–8677, Japan; 9 Department of Rheumatology, National Hospital Organization Nagasaki Medical center, Nagasaki 380–8582, Japan; 10 Department of Clinical Oncology, Ehime Graduate School of Medicine, Ehime 791–0295, Japan; 11 Department of Rheumatology, Japan Community Healthcare Organization, Isahaya General Hospital, Nagasaki 854–8501, Japan; 12 Department of Gastroenterology, Rheumatology and Clinical Immunology, Sapporo Medical University School of Medicine, Hokkaido 060–8543, Japan; 13 Medical Informatics Division and Department of Internal Medicine, Gastroenterology, Shinshu University School Hospital, Nagano 390–8621, Japan; 14 First Department of Internal Medicine, Department of Immunology and Rheumatology, Nagasaki Graduate School of Health Sciences, Nagasaki 852–8520, Japan; 15 First Department of Internal Medicine, School of Medicine, University of Occupational and Environmental Health, Japan, Fukuoka 807–8555, Japan; 16 Department of Internal Medicine, Faculty of Medicine, University of Tsukuba, Ibaraki 305–8575, Japan; 17 Division of Nephrology, Mitoyo General Hospital, Kagawa 769–1695, Japan; 18 Department of Clinical Immunology, Graduate School of Medicine and Faculty of Medicine, Kyoto University, Kyoto 606–8501, Japan; University of Jaén, SPAIN

## Abstract

**Background:**

IgG4-related disease (IgG4-RD) is a new clinical entity of unknown etiology characterized by elevated serum IgG4 and tissue infiltration by IgG4-positive plasma cells. Although aberrancies in acquired immune system functions, including increases in Th2 and Treg cytokines observed in patients with IgG4-RD, its true etiology remains unclear. To investigate the pathogenesis of IgG4-RD, this study compared the expression of genes related to innate immunity in patients with IgG4-RD and healthy controls.

**Materials and Methods:**

Peripheral blood mononuclear cells (PBMCs) were obtained from patients with IgG4-RD before and after steroid therapy and from healthy controls. Total RNA was extracted and DNA microarray analysis was performed in two IgG4-RD patients to screen for genes showing changes in expression. Candidate genes were validated by real-time RT-PCR in 27 patients with IgG4-RD and 13 healthy controls.

**Results:**

DNA microarray analysis identified 21 genes that showed a greater than 3-fold difference in expression between IgG4-RD patients and healthy controls and 30 genes that showed a greater than 3-fold change in IgG4-RD patients following steroid therapy. Candidate genes related to innate immunity, including those encoding Charcot–Leyden crystal protein (CLC), membrane-spanning 4-domain subfamily A member 3 (MS4A3), defensin alpha (DEFA) 3 and 4, and interleukin-8 receptors (IL8R), were validated by real-time RT-PCR. Expression of all genes was significantly lower in IgG4-RD patients than in healthy controls. Steroid therapy significantly increased the expression of DEFA3, DEFA4 and MS4A3, but had no effect on the expression of CLC, IL8RA and IL8RB.

**Conclusions:**

The expression of genes related to allergy or innate immunity, including CLC, MS4A3, DEFA3, DEFA4, IL8RA and IL8RB, was lower in PBMCs from patients with IgG4-RD than from healthy controls. Although there is the limitation in the number of patients applied in DNA microarray, impaired expression of genes related to innate immunity may be involved in the pathogenesis of IgG4-RD as well as in abnormalities of acquired immunity.

## Introduction

IgG4-related disease (IgG4-RD) is a new emerging disease entity characterized by elevated serum IgG4 concentrations and tissue tumefaction or infiltration by IgG4-positive plasma cells [1, 2]. Clinically, IgG4-RD is characterized by a general inflammatory state as well as manifestations specific to individual affected organs, including the lacrimal glands, salivary glands, pancreas, bile duct, lungs, liver, kidneys, prostate, thyroid, retroperitoneum, arteries, lymph nodes, skin, central nervous system, and breasts. Most patients with IgG4-RD experience multiple organ involvement, either synchronously or metachronously, whereas others show only a single site of involvement [1, 2]. IgG4-RD occurs more frequently in older adults than in younger individuals (median age, 58 years). Once it occurs, it slowly progresses and is characterized by elevated serum IgE [3] and relatively weak indicators of inflammation, such as low titer of CRP[4]. Steroid therapy has been found effective in most patients [3, 5].

IgG4-RD is also characterized by several aberrant findings in the acquired immune system. For example, the numbers of CD4^+^CD25^+^Foxp3^+^ regulatory T cells (Tregs) in affected tissues and peripheral blood are significantly higher in patients with IgG4-RD than in healthy controls [6–8]. In addition, several autoantibodies, including anti-carbonic anhydrase II and anti-lactoferrin, are often present in patients with IgG4-RD, especially those with IgG4-related autoimmune pancreatitis (AIP) [9, 10]. Furthermore, the expression of Th2 and Treg cytokines is dominant in IgG4-RD [6, 11, 12]. At present, however, it is not clear whether IgG4-RD is caused by abnormalities in acquired immunity like autoimmune diseases, or whether the excess production of IgG4 is a true cause of IgG4-RD or an epiphenomenon associated with inflammatory and/or allergic reactions.

Although its true etiology remains unclear, infections with various pathogens, including Helicobacter pylori [13, 14], gram-negative bacteria [15] and Mycobacterium tuberculosis [16], have been reported in patients with IgG4-RD. These pathogens may induce the production of IgG4, which, in turn, may block activation of the innate immune system by inhibiting the activities of IgG1 and the formation of immune complexes, resulting in the persistence of these infections [17]. We therefore attempted to identify genes of the innate immune system that are related to the pathogenesis or clinicopathology of IgG4-RD.

Initially, we utilized DNA microarray analysis to select candidate genes with levels of expression three times higher or lower in patients with IgG4-RD than in healthy controls. Subsequently we compared expression of genes in patients with IgG4-RD before and after steroid treatment to identify genes up- and down regulated by steroids. Finally, we performed transcriptome analysis of PBMCs from 27 patients with IgG4-RD and 13 healthy controls to validate the significance of these genes.

## Materials and Methods

### Patients and samples

IgG4-RD was diagnosed according to the comprehensive diagnostic criteria for IgG4-RD [18]. These patients were registered in the research project of the Research Program for Intractable Disease of the Ministry of Health, Labor, and Welfare (MHLW) of Japan, designed to establish diagnostic criteria for IgG4-related multi-organ lymphoproliferative syndrome (IgG4-MOLPS). Initial steroid therapy for IgG4-RD consisted of prednisolone (0.6 mg/kg body weight per day), with this dose reduced 10% every 2 weeks. Two subjects with characteristic clinical features of IgG4-RD, including extreme elevation of serum IgG4 (5630 and 2950 mg/dl, respectively) and multiple organs showing tumefaction by IgG4-positive plasma cells, including the salivary glands, duodenum, lymph nodes, bile ducts, pancreas and prostate, are described in Table 1. The IgG, IgG4, and IgE concentrations in all healthy controls were within normal ranges.

**Table 1:** Profiles of IgG4-RD patients analyzed by DNA microarrays.

No		Age	Sex	IgG	Post Tx. IgG	IgG4	Post Tx. IgG4	IgE	Post Tx. IgE	Lesions
1	IgG4-RD	66	M	5,630	464	3,120	291	265.0	51.5	SG, DD, LN
2	IgG4-RD	63	M	2,950	638	1,540	184	7.9	5.0	SG, BD, PC, PS
3	Healthy	57	M	-	-	-	-	-	-	-
4	Healthy	58	M	-	-	-	-	-	-	-
5	Healthy	62	M	-	-	-	-	-	-	-
6	Healthy	64	M	-	-	-	-	-	-	-

Patients were diagnosed according to the comprehensive diagnostic criteria for IgG4-RD [6] and were subsequently treated with steroids. IgG(mg/dL), IgG4(mg/dL), IgE(IU/mL). Abbreviations: SG, salivary gland; DD, duodenum; LN, lymph node; BD, bile duct; PC, pancreas; PS, prostate.

For DNA microarray, heparinized peripheral blood was obtained from these two IgG4-RD patients before and 3 months after starting steroid therapy and from four healthy normal controls (all men, median age 59 years). For validation assays, heparinized peripheral blood was obtained from 27 patients with IgG4-RD (19 men, 8 women; median age 66 years) before steroid therapy and from 13 healthy controls (9 men, 4 women; median age 61 years). Peripheral blood was also obtained from 20 patients with IgG4-RD three to six months after commencement of steroid treatment. Subject characteristics are shown in Table 2.

**Table 2:** Profiles of IgG4-RD patients assayed by Real time RT-PCR.

No	Age	Sex	IgG (mg/dL)	IgG4 (mg/dL)	IgG4(postTx)	IgE (IU/mL)	Lesion
1*	66	M	5,630	3,120	291	265	SG, DD, LN
2*	63	M	2,950	1,540	184	7	SG, BD, PC, PS
3	69	F	1,950	362	44	151	SG
4	62	M	1,500	435	89	301	SG, PC, LN
5	60	F	1,150	110	12	399	LG, SG, PC
6	79	M	4,020	1,460	98	330	SG, RF, KN, PS, LN
7	70	M	2,563	1,160	326	283	SG, RF, IP, UR
8	65	M	6,786	3,880	447	673	PS, LN
9	72	M	2,980	254	9	85	PL, RF, PC, PG, LN
10	73	M	3,377	1,770	690	1,216	SG, PC
11	47	F	1,365	304	35	238	LG, SG
12	66	M	1,679	756	153	631	SG, IP
13	53	M	1,692	313	55	494	LG, UR
14	70	M	2,090	314	201	190	LG, SG
15	52	F	3,038	1,300	298	327	LG, SG
16	38	M	2,861	1,440	315	219	LG, IP
17	66	F	3,214	1,370	174	60	LG, SG, IP, BD
18	66	F	4,174	1,300	179	100	LG, SG, RF, IP, LN
19	59	M	1,603	499	124	1,139	RF
20	59	M	2,456	1,430	226	262	PC, IP
21	74	M	3,250	788		189	AA, LN
22	91	M	4,577	669		626	SG
23	55	M	3,087	1,760		837	PS, IP
24	57	F	2,442	990		1,181	SG
25	68	F	1,620	419		159	SG, PC
26	71	M	1,800	373		180	SG, PC, LN
27	56	M	4,010	2,160		680	LG, SG, LN

Real time RT-PCR was performed on mRNA samples isolated from the PBMCs of 27 patients (19 men, 8 women; median age 66 years) with IgG4-RD. Case 1* and 2* are the same patients in Table 1. Patients with steroid treatment are Case1 to Case 20, and IgG4 (post Tx) means the value of serum IgG4 after steroid treatment. Abbreviations: SG, salivary gland; DD, duodenum; LN, lymph node; BD, bile duct; PC, pancreas; PS, prostate; AA, aorta abdominalis; IP, interstitial pneumonia; LG, lacrimal gland; RF, retroperitoneal fibrosis; KN, kidney; UR, ureter; PL, pleura; PG, pituitary gland.

This study was approved by the institutional ethics board of each institution; Kanazawa Medical University, Kanazawa University, Gunma University Graduate School of Medicine, University of Toyama, Nagaoka Red Cross Hospital, Kansai Medical University, Tokyo Metropolitan Komagome Hospital, National Hospital Organization Nagasaki Medical center, Ehime Graduate School of Medicine, Isahaya Health Insurance General Hospital, Sapporo Medical University School of Medicine, Shinshu University School Hospital, Nagasaki Graduate School of Health Sciences, University of Occupational and Environmental Health, University of Tsukuba, Mitoyo General Hospital, and Kyoto University. Informed consent for publication of all data and samples was obtained from each patient. The research was conducted in compliance with the Declaration of Helsinki.

### Isolation of total RNA

Immediately after blood collection, PBMCs were separated using Lymphoprep (Axis-Shield, Oslo, Norway), according to the manufacturer’s instructions. Total RNA was extracted using RNeasy Plus Mini kits (Qiagen, Hilden, Germany), according to the manufacturer’s protocol. The concentration and quality of these RNA samples were assessed by measuring UV absorbance at 260 and 280 nm (A_260_/_280_ ratio) and by images of 18S and 28S ribosomal bands in agarose gel electrophoresis.

### DNA microarrays

To exclude any gender-related differences in gene expression, DNA microarray analysis was performed only on samples obtained from male patients and controls. Total RNA was reverse transcribed to cDNA using Ambion WT Expression kits (Applied Biosystems, Foster City, CA), labeled with GeneChip WT Terminal Labeling and Controls kits (Affymetrix, Santa Clara, CA), and hybridized to GeneChip Human Gene 1.0 ST Arrays (Affymetrix), which include 28869 probes. Digitalized image data were processed using GeneChip Operating Software (Affymetrix). Following background correction and 50^th^ percentile normalization, the microarray results were analyzed using GeneSpring version 11.0 software (Agilent Technologies, Santa Clara, CA). The microarray expression data discussed in this paper (S1 Table) have been deposited in NCBI’s Gene Expression Omnibus (GEO) and are accessible with GEO Series accession number GSE66465 (http://www.ncbi.nlm.nih.gov/geo/query/acc.cgi?acc=GSE66465).

Genes showing ≥ 3-fold differences in expression between patients and healthy controls, and before and after steroid therapy in IgG4-RD patients, were selected, and statistically processed by K-means clustering. In addition to clustering, average fold changes were determined to screen for genes with altered levels of expression. Gene pathway databases were assessed by Ingenuity Pathways Analysis (Ingenuity Systems, Redwood City, CA) [19, 20].

### Real-time PCR

Total RNA from PBMCs was reverse transcribed to cDNA using Sensiscript RT kits (Qiagen, Hilden, Germany) and oligo (dT) primers according to the manufacturer’s instructions. Primers and TaqMan probes were purchased from Applied Biosystems. Real-time PCR was performed with an ABI Prism 7700 Sequence Detector (Applied Biosystems), using a TaqMan gene expression assay (Applied Biosystems) and Thunderbird Probe qPCR mix (Toyobo, Osaka, Japan). The relative quantity of each target mRNA was normalized relative to that of the internal control, β-actin.

### Statistical analyses

In real-time PCR analysis, between-group comparisons were performed using the Mann—Whitney U-test or Student’s *t* test. All statistical analyses were performed using Stat View version 5.0. In all analyses, *P* < 0.05 was defined as statistically significant.

## Results

### DNA microarray analysis

DNA microarray analysis was performed to identify candidate genes that may be involved in IgG4-RD pathogenesis (S1 Table). Total RNA was prepared from PBMCs of two patients with IgG4-RD (Table 1) and from four healthy controls and reverse transcribed. Genes showing ≥3-fold differences in expression between patients, regardless of steroid treatment, and controls were identified, inasmuch as they may be congenitally altered in patients with IgG4-RD and may be responsible for the pathogenesis of this disease. The average values of the four data sets from the two patients with IgG4-RD before and after steroid treatment, and of the four data sets from healthy volunteers, were classified by K-means clustering followed by separation based on 3-fold changes in level of expression. This method identified 21 genes that showed ≥ 3-fold differences in level of expression between IgG4-RD patients (both before and after steroid treatment) and healthy controls (Table 3). Five of these genes were decreased in IgG4-RD patients, including those encoding Charcot—Leyden crystal protein (CLC), desmocollin1 (DSC1), interleukin 8 receptors alpha (IL8RA) and beta (IL8RB), and leucine rich repeat neuronal 3 (LRRN3), whereas 16 were increased in IgG4-RD (Table 3). Ingenuity Pathways Analysis (Ingenuity Systems) [19, 20] confirmed that no reported changes in expression of these genes were associated with steroid treatment (data not shown).

**Table 3:** Genes showing ≥3-fold differences in expression level in IgG4-RD patients and healthy controls.

Transcripts Cluster Id	Regulation	Gene symbol	Gene description
* **8036755**	**down**	**CLC**	**Charcot-Leyden crystal protein**
8022728	down	DSC1	desmocollin 1
* **8058905**	**down**	**IL8RA**	**interleukin 8 receptor, alpha**
* **8048227**	**down**	**IL8RB**	**interleukin 8 receptor, beta**
8135488	down	LRRN3	leucine rich repeat neuronal 3
7981708	up	IGHE	immunoglobulin heavy constant epsilon
8095736	up	AREG|LOC727738	amphiregulin (schwannoma-derived growth factor)
8095744	up	AREG|LOC727738	amphiregulin (schwannoma-derived growth factor)
8101322	up	MOP-1	
8055952	up	NR4A2	nuclear receptor subfamily 4, group A, member 2
8156848	up	NR4A3	nuclear receptor subfamily 4, group A, member 3
8012349	up	PER1	period homolog 1 (Drosophila)
7908388	up	RGS1	regulator of G-protein signaling 1
8005547	up	SNORD3A	small nucleolar RNA, C/D box 3A
8005553	up	SNORD4A	small nucleolar RNA, C/D box 4A
8013323	up	SNORD5A	small nucleolar RNA, C/D box 5A
8013325	up	SNORD6A	small nucleolar RNA, C/D box 6A
8013329	up	SNORD7A	small nucleolar RNA, C/D box 7A
7922416	up	SNORD75	small nucleolar RNA, C/D box 75
7982597	up	THBS1	thrombospondin 1
8116992	up	UNQ9364	FLFF9364

We identified 21 genes showing a ≥3-fold increase (16 genes) or decrease (5genes) in expression level among 4 samples from 4 healthy controls and 2 IgG4-RD patients before and after therapy.

*processed to the validation.

To identify genes affected by steroid treatment, transcriptomes in IgG4-RD patients were compared before and after steroid therapy. Average values from two typical IgG4-RD patients with extreme elevation of serum IgG4 and multiple organ involvement (Table 1) and from four healthy volunteers were compared. Thirty-six genes showed ≥ 3-fold differences in expression before and after steroid therapy of IgG4-RD patients (Table 4). For example, steroid therapy decreased the expression of IFI44L, SNORA42, and HIST1H2BB, while increasing the expression of the other genes including membrane-spanning 4-domains, subfamily A, member 3 (MS4A3), defensin alpha 3 (DEFA3) and alpha 4 (DEFA4). K-means clustering, used for statistical processing of disease-associated genes showing lower expression prior to steroid treatment and higher expression after treatment, identified 30 genes, all of which were increased ≥3-fold following steroid therapy (Table 5). These genes may be markers of patient recovery, because their levels of expression correlated with steroid treatment.

**Table 4:** Genes showing ≥3-fold changes in expression in IgG4-RD patients in response to steroid therapy.

Transcripts	Cluster ID	Case1 FC	Case 2 FC	Gene symbol	Gene description
**Decrease**					
	7902541	6.073982	3.3045993	IFI44L	interferon-induced protein 44-like
	7920873	5.086785	3.571676	SNORA42	small nucleolar RNA, H/ACA box 42
	8124394	4.4283495	6.986072	HIST1H2BB	histone cluster 1, H2bb
**Increase**					
	7922976	4.3904357	3.8047059	PTGS2	prostaglandin-endoperoxide synthase 2
	7933872	4.3444343	4.182845	EGR2	early growth response 2
	* **7940216**	**27.282946**	**22.44819**	**MS4A3**	**membrane-spanning 4-domains, subfamily A, member 3**
	7948444	5.2985225	6.0289493	TCN1	transcobalamin I
	7951246	35.18979	20.34096	MMP8	matrix metallopeptidase 8
	7969288	44.24184	3.3508606	OLFM4	olfactomedin 4
	7973105	6.7971625	5.0625668	RNASE3	ribonuclease, RNase A family, 3
	7978351	11.790634	3.3185043	CTSG	cathepsin G
	7995237	8.0480795	14.685388	ERAF	erythroid associated factor
	8015991	3.9202752	5.3016458	SLC4A1	solute carrier family 4, anion exchanger, member 1
	8016932	8.801972	4.1204348	MPO	myeloperoxidase
	8021645	4.3249626	4.259984	SERPINB10	serpin peptidase inhibitor, clade B (ovalbumin), member 10
	8029098	15.656315	7.720778	CEACAM6	carcinoembryonic antigen-related cell adhesion molecule 6
	8036755	4.2519445	13.180141	CLC	Charcot-Leyden crystal protein
	8037222	23.474125	13.198722	CEACAM8	carcinoembryonic antigen-related cell adhesion molecule 8
	8037298	8.516854	8.1878	LOC100130904	similar to CD177 molecule
	8054722	7.441816	8.529232	IL1B	interleukin 1, beta
	8062444	8.891481	11.64061	BPI	bactericidal/permeability-increasing protein
	8066493	6.6776667	5.0690618	SLPI	secretory leukocyte peptidase inhibitor
	8086607	6.1559343	4.076412	LTF	lactotransferrin
	8100994	7.2271314	3.2157035	CXCL2	chemokine (C-X-C motif) ligand 2
	8122058	5.4273343	3.2703066	ARG1	arginase, liver
	8126905	10.53019	13.541852	CRISP3	cysteine-rich secretory protein 3
	8145281	5.086257	4.649192	SLC25A37	solute carrier family 25, member 37
	8145291	4.369647	3.5463853	SLC25A37	solute carrier family 25, member 37
	* **8149109**	**27.479057**	**15.6270685**	**DEFA4**	**defensin, alpha 4, corticostatin**
	8149116	69.414474	54.943527	DEFA1	defensin, alpha 1
	8149126	69.360466	54.966377	DEFA1	defensin, alpha 1
	* **8149137**	**69.42058**	**55.047955**	**DEFA3**	**defensin, alpha 3,**
	8151592	7.4683266	22.69821	CA1	carbonic anhydrase I
	8158167	6.812407	3.5723207	LCN2	lipocalin 2
	8173135	5.2392645	9.046565	ALAS2	aminolevulinate, delta-, synthase 2
	8177222	4.341619	5.943259	CD24	

We identified 36 genes showing ≥3-fold increase or decrease in expression in IgG4-RD patients in response to steroid therapy. IFI44L, SNORA42 and HIST1H2BB had decreased expression by steroid therapy, and the other genes increased. Abbreviations: FC, fold changes.

*processed to the validation.

**Table 5:** The list of disease-associated genes using K-means clustering.

Transcripts Cluster ID	Gene symbol	Gene description
* **7940216**	**MS4A3**	**membrane-spanning 4-domains, subfamily A, member 3 (hematopoietic cell-specific)**
7946033	HBB	hemoglobin, beta
7948444	TCN1	transcobalamin I (vitamin B12 binding protein, R binder family)
7951246	MMP8	matrix metallopeptidase 8 (neutrophil collagenase)
7969288	OLFM4	olfactomedin 4
7973105	RNASE3	ribonuclease, RNase A family, 3 (eosinophil cationic protein)
7978351	CTSG	cathepsin G
7991762	HBA1|HBA2	hemoglobin, alpha 1 | hemoglobin, alpha 2
7991766	HBA1|HBA2	hemoglobin, alpha 1 | hemoglobin, alpha 2
7995237	ERAF	erythroid associated factor
8015991	SLC4A1	solute carrier family 4, anion exchanger, member 1 (erythrocyte membrane protein band 3, Diego blood group)
8016932	MPO	myeloperoxidase
8021645	SERPINB10	serpin peptidase inhibitor, clade B (ovalbumin), member 10
8029098	CEACAM6	carcinoembryonic antigen-related cell adhesion molecule 6 (non-specific cross reacting antigen)
8037222	CEACAM8	carcinoembryonic antigen-related cell adhesion molecule 8
8037298	LOC100130904	similar to CD177 molecule
8062444	BPI	bactericidal/permeability-increasing protein
8066493	SLPI	secretory leukocyte peptidase inhibitor
8086607	LTF	lactotransferrin
8122058	ARG1	arginase, liver
8126905	CRISP3	cysteine-rich secretory protein 3
8145281	SLC25A37	solute carrier family 25, member 37
8145291	SLC25A37	solute carrier family 25, member 37 | hypothetical protein LOC100133914
* **8149109**	**DEFA4**	**defensin, alpha 4, corticostatin**
8149116	DEFA1	defensin, alpha 1 defensin, alpha 3, neutrophil-specific
8149126	DEFA1	defensin, alpha 1 defensin, alpha 3, neutrophil-specific
* **8149137**	**DEFA3**	**defensin, alpha 3, neutrophil-specific defensin, alpha 1 |defensin, theta 1 pseudogene**
8151592	CA1	carbonic anhydrase I
8173135	ALAS2	aminolevulinate, delta-, synthase 2
8177222	CD24	

K-means clustering was used for statistical processing of disease-associated genes, we identified 30 genes. All genes had increased expression level by steroid therapy.

*processed to the validation.

### Real-time PCR validation

**Fig 1:**
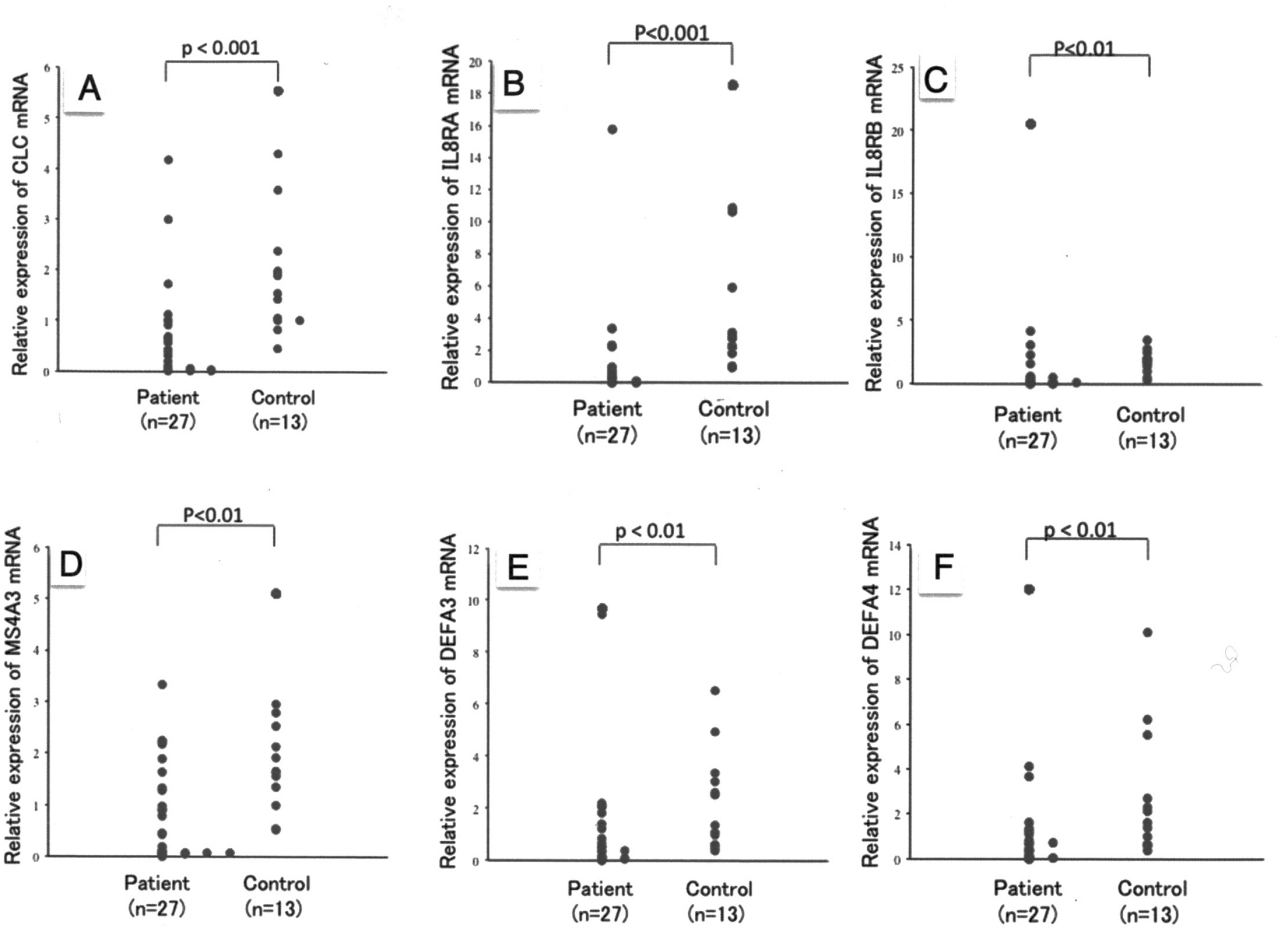
Comparison of gene expression between patients with IgG4-RD and healthy controls. Relative expression of genes in PBMCs from 27 patients with IgG4-RD and 20 healthy controls. (A) CLC; Charcot—Leyden crystal protein. (B) IL8RA; interleukin 8 receptor alpha. (C) IL8RB; interleukin 8 receptor beta. (D) MS4A3; membrane-spanning 4-domain subfamily A member 3. (E) DEFA3; defensin alpha 3. (F) DEFA4; defensin alpha 4. Expression of all genes was significantly lower in PBMCs from untreated IgG4-RD patients than from healthy controls (p< 0.01).

Infections with various pathogens, including *Helicobacter pylori* [13, 14], gram-negative bacteria [15] and *Mycobacterium tuberculosis* [16], have been reported in patients with IgG4-RD. Therefore, we selected, from among the genes identified by DNA microarray analysis, several related to innate immunity, including those encoding CLC, also called galectin 10, IL8RA and IL8RB (Table 3), membrane-spanning 4-domains, subfamily A, member 3 (MS4A3) and defensins alpha 3 (DEFA3) and 4 (DEFA4) (Tables 4 and 5), and performed real-time RT-PCR to assess their levels of expression. We found that the levels of expression of CLC, MS4A3, DEFA3, DEFA4, IL8RA and IL8RB mRNAs were all lower in IgG4-RD patients than in healthy controls (Fig 1).

**Fig 2:**
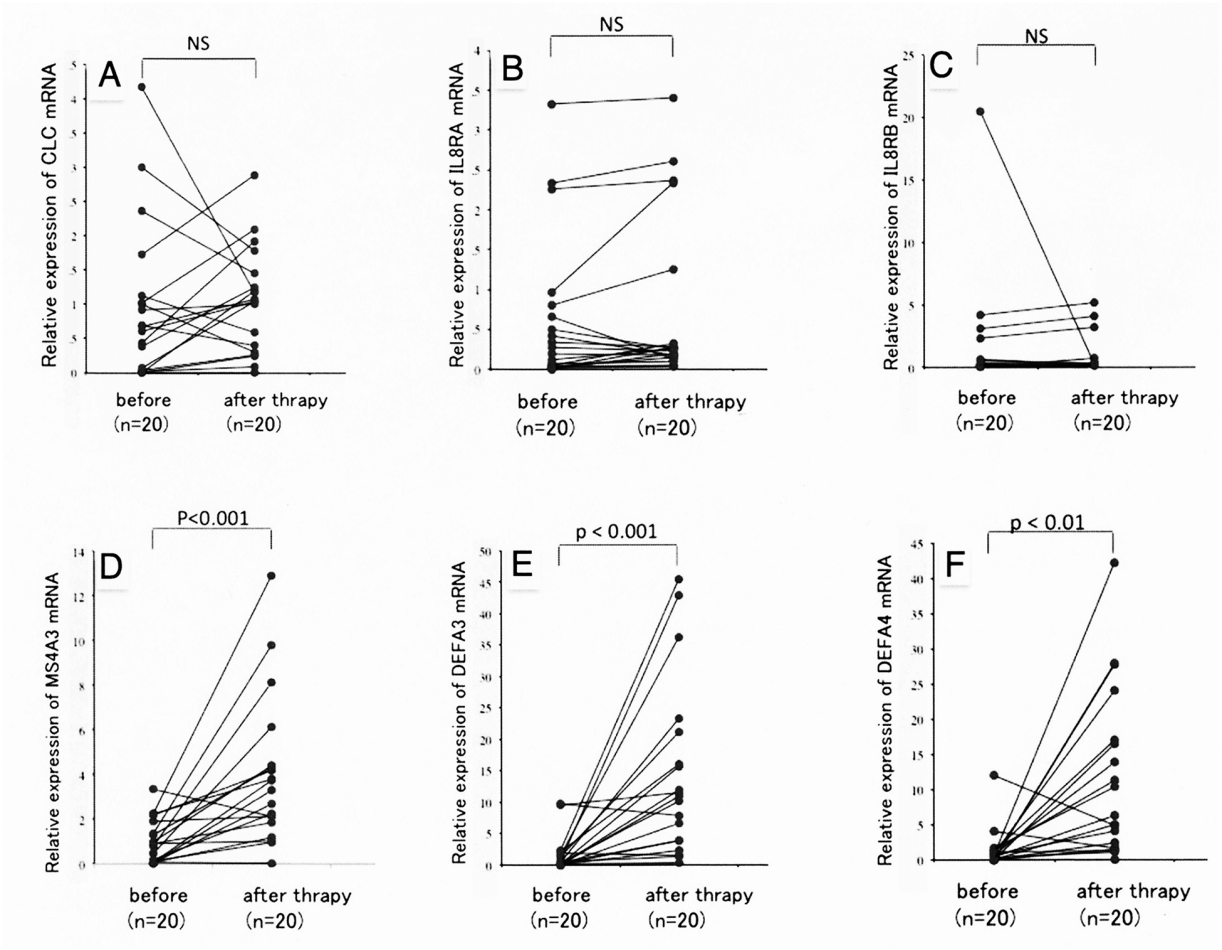
Gene expression in PBMCs from 20 patients with IgG4-RD, before and after steroid treatment. (A) CLC; Charcot—Leyden crystal protein. (B) IL8RA; interleukin 8 receptor alpha. (C) IL8RB; interleukin 8 receptor beta. (D) MS4A3; membrane-spanning 4-domain subfamily A member 3. (E) DEFA3; defensin alpha 3. (F) DEFA4; defensin alpha 4. Levels of CLC, IL8RA and IL8RB mRNA were not altered in IgG4-RD patients by steroid therapy (A-C), whereas those of MS4A3, DEFA3 and DEFA4 were significantly increased following steroid therapy (D-F, p<0.01).

We also assessed the effects of steroid therapy on the levels of expression of these genes. Steroid therapy had no effects on the expression levels of the genes encoding CLC, IL8RA and IL8RB (Fig 2A–2C), but significantly increased the levels of expression of the genes encoding MS4A3, DEFA3 and DEFA4 (Fig 2D–2F).

## Discussion

Elevated serum IgG4 concentration and tissue infiltration by IgG4-positive cells are key events in IgG4-RD. IgG4 itself may play anti-inflammatory rather than proinflammatory roles due to its unique structure and functions. For example, the interactions of IgG4 with the Fcγ receptor and C1q are weaker than those of the other immunoglobulin subclasses [21]. Moreover, IgG4 antibodies can exchange Fab arms by swapping a heavy chain and its attached light chain [22], thus functioning as bispecific, as well as monospecific, molecules. These properties may protect against type I allergy by inhibiting IgE function, and may prevent type II and III allergies in patients with autoimmune diseases by blocking the Fc-mediated effector functions of IgG1 and inhibiting the formation of large immune complexes [21, 22].

It is not clear, however, whether IgG4 itself is the major factor involved in the pathogenesis of IgG4-RD. Efforts have therefore been made to identify more important upstream pathogenetic changes. Abnormalities in the acquired immune system have been observed in patients with IgG4-RD, such as increased numbers of Tregs in peripheral blood and focal lesions, including the organs involved in IgG4-RD, increases associated with the etiopathogenesis of IgG4-RD [7, 8]. Increases in Tregs have been associated with increased production of Th2 cytokines, especially IL-10, which increases IgG4 production by B cells, and TGF-β, which induces the characteristic fibrotic features of IgG4-RD [6]. However, the mechanisms responsible for the increases in Tregs and Th2 cytokines remain unclear. We therefore attempted to identify genes that may be associated with disease etiology or pathogenesis by DNA microarray analysis of PBMCs from two patients with IgG4-RD (S1 Table).

Among the genes showing ≥3-fold differences in expression between IgG4-RD patients and controls, we selected CLC because of its clinical association with type I hypersensitivity such as bronchial asthma [23, 24]. Furthermore, CLC protein has been reported to be a marker of chemoattractant receptor homologous molecule expressed on T-helper type 2 cells (CRTH2), a prostaglandin D2 receptor [25], and CLC expression has been observed in the cytoplasm of CD4^+^CD25^+^ Tregs, with little expression in CD4^+^CD25^-^T cells [26]. We found that the level of CLC mRNA was significantly lower in IgG4-RD patients than in controls (Fig 1), despite the serum IgE concentrations being higher in IgG4-RD patients (Table 2). These findings suggested that the elevated serum IgE observed in IgG4-RD patients may not be due to type I hypersensitivity.

In addition, inhibition of CLC expression in Tregs in vitro has been reported to lead to the proliferation of CD4^+^ T cells when co-cultured with Tregs, as well as augmenting the proliferation of the Tregs themselves. Furthermore, transfection of CLC siRNA into Tregs increased IFN-γ and TNF-α production, while having no effect on surface markers and transcription factors, such as CD25, CTLA-4, CD45RO, CD62L, and Foxp3 [26]. A mutation in the Foxp3 gene was found to lead to immune dysregulation, polyendocrinopathy, enteropathy, and X-linked (IPEX) syndrome in humans, and to various autoimmune, inflammatory, and allergic conditions in scurfy mice, with lymphocyte infiltration into multiple organs, and the development of hyper-IgE-emia [27, 28]. The phenotypes of mice with abnormal Treg functions were similar to those of patients with IgG4-RD. Thus, a decrease in Treg function resulting from the reduced expression of CLC, despite the increase in number of Tregs, may be related to the etiopathogenesis of IgG4-RD. We also found that the levels of expression of the IL-8 receptors, IL8RA and IL8RB, were significantly lower in untreated IgG4-RD patients than in healthy controls (Fig 1), with steroid therapy having no effect in the former (Fig 2). Stimulation by inflammatory cytokines, such as IL-1, tumor necrosis factor (TNF)-α, and IL-8, induces chemokine production by monocytes and macrophages. These chemokines play a major role in the innate immune system, by promoting neutrophil migration and activation. Since neutrophil migration is abrogated at the site of inflammation in IL-8R knockout mice [29, 30], decreased expression of IL8RA and IL8RB may be involved in impaired innate immune system.

As IgG4 itself may play anti-inflammatory roles due to its unique properties, we selected three genes showing increased expression in IgG4-RD patients after steroid therapy, i.e. MS4A3, DEFA3 and DEFA 4. MS4A3, also called HTm4, belongs to the MS4A family, which includes CD20 (MS4A1) and Fcε RI antigen receptor β-chain (MS4A2). These proteins have four transmembrane domains in their N-terminal regions and act as cell surface signaling molecules and intercellular proteins, as well as having C-terminal cytoplasmic regions [31]. Among cells of the hematopoietic system, basophils show the highest level of MS4A3 expression, with other granulocytes and B and T cells also showing expression at lower levels [31]. Chromosomally, the MS4A3 gene is located adjacent to the gene encoding Fcε RI antigen receptor β-chain (MS4A2), which is thought to be involved in type I allergic reactions [32]. Although MS4A3 was thought to be associated with elevated serum IgE and allergic rhinitis, we found that its level of expression was significantly lower in PBMCs from IgG4-RD patients prior to steroid treatment than from healthy controls (Fig 1), and that its level of expression in IgG4-RD patients increased after steroid treatment (Fig 2). Although expression of MS4A3 may be diagnostic of IgG4-RD, further studies are required to determine their association.

We also found that the expression of the DEFA3 and DEFA4 genes was lower in patients with IgG4-RD than in controls (Fig 1). Defensin is a representative antibacterial peptide in mammals, with its antibacterial activity functioning as an effector in the innate immune system [33]. Human neutrophils express both α- and β-defensin, with 5%-7% of the total protein in these cells being human neutrophil peptides-3 and -4, which are encoded by the DEFA3 and DEFA4 genes, respectively, with these proteins also being present in azurophil granules [33]. Moreover, defensin is produced not only by neutrophils, but by phagocytic cells, lymphocytes and epithelial cells [34]. In addition, α-defensin acts to mobilize dendritic and T cells to sites of bacterial invasion, thereby serving as an intermediary between the innate and acquired immune systems [33, 35].

Our analysis of gene expression in response to steroid treatment showed that the levels of expression of genes encoding bactericidal substances, such as myeloperoxidase, cathepsin G, bactericidal/permeability-increasing protein and lactotransferrin, were lower in IgG4-RD patients prior to steroid treatment than in healthy controls (Table 5). Our findings, that the levels of expression of α-and β-defensin (Table 5) and of IL8RA and IL8RB (Table 3) were lower in PBMCs from IgG4-RD patients than from controls, suggest that functions of innate immunity may be impaired in IgG4-RD patients. Thus impaired transition from innate to acquired immunity may be related to the etiopathogenesis of IgG4-RD.

Assessment of tissue lesions from patients with IgG4-RD showed that the levels of expression of the Th1 cytokine, IFN-γ, and the Th2 and inhibitory cytokines, IL-10 and transforming growth factor-β (TGF-β), were all elevated compared with healthy controls [6]. Our gene cluster analysis, however, did not find ≥ 3-fold differences in the levels of expression of inhibitory cytokines associated with Treg production and function, such as TGF-β and IL-10. In contrast to the previous study, we analyzed mRNA levels in PBMCs of IgG4-RD patients, not in the lesions themselves. Therefore, the discrepancy between studies may reflect secondary changes occurring during the disease process. It is also known that RNA expression and protein levels are not always correlated in cells or the circulation. Since there is the limitation of our analysis that only two patients with IgG4-RD have been analyzed by DNA microarray, our results, showing decreased expression of innate immune system-related genes, will require further mechanistic studies.

To our knowledge, however, no previous reports have measured serum IL-10 and TGF-β concentrations in IgG4-RD patients. Thus, our results suggest that the levels of inhibitory cytokines are not increased in the peripheral blood of IgG4-RD patients.

## Conclusions

We found that the levels of expression of genes involved in allergy development and innate immunity, including those encoding CLC, MS4A3, DEFA3, DEFA4, IL8RA, and IL8RB, were lower in PBMCs from IgG4-RD patients than from healthy controls. These findings suggest that impairments in the innate immune system may be responsible, at least in part, for the pathogenesis of IgG4-RD. Stimulation of nucleotide-binding oligomerization domain (NOD)-like receptors (NLR) on monocytes was found to increase IgG4 production by B cells [36]. Moreover, monocytes from patients with IgG4-RD showed greater IgG4 production by B cells than monocytes from healthy individuals upon stimulation with NOD-2 ligand [36]. Although the mechanisms of IgG4 production and their contribution to IgG4-RD are still unclear, cross-talk between the innate and acquired immune system may play key roles in the pathogenesis of IgG4-RD [17].

## Supporting Information

S1 Table DNA microarray analysis of two typical patients with IgG4-RD. Total RNA was prepared from PBMCs of two patients with IgG4-RD (Table 1) and from four healthy controls and reverse transcribed.
(XLS)
